# Ethanol and Cancer Induce Similar Changes on Protein Expression Pattern of Human Fibroblast Cell

**Published:** 2016

**Authors:** Mona Zamanian–Azodi, Mostafa Rezaei-Tavirani, Sara Rahmati-Rad, Majid Rezaei Tavirani

**Affiliations:** a *Proteomics Research Center, Shahid Beheshti University of Medical Sciences, Tehran, Iran.*; b *Department of Cell and Molecular Biology, Faculty of Science, University of Tehran, Tehran, Iran. *; c *Faculty of Medicine, Ilam University of Medical Sciences, Ilam, Iran.*

**Keywords:** Ethanol, Human Foreskin Fibroblast cell line (HFFF2), Proteomics, Gene Ontology, Protein-protein Interaction (PPIs)

## Abstract

Ethanol has a vast consumption around the world. Many researches confirmed some adverse effect of this component on human health. In addition, recent studies showed significant alteration in both cellular population, and protein profile of human foreskin fibroblast cell line (HFFF2) in the specific dosage of ethanol. Here, the role and interaction of some proteins (characterized by significant alteration in expression due to ethanol effect) analyzed by proteomics and evaluated by considering cancerous case. 2D-electrophoresis findings of comparison of normal fibroblast cells and treated fibroblast with 270 mM dosage of ethanol analyzed by using SameSpots software, R software, and Cytoscape for protein-protein interaction (PPI) investigation. Six proteins with significantly altered expression associated with fundamental properties in a cell identified in ethanol-treated sample. These include AnnexinA5, Heterogeneous nuclear ribonucleoprotein A1, Rho-GDP dissociation inhibitor, Cathepsin L, Cu/Zn-SOD, Rho-GDP dissociation inhibitor, and Serpin peptidase inhibitor. Surprisingly, all these proteins were down-regulated and this pattern is similar to nasopharyngeal carcinoma-associated stromal fibroblast sample. Additionally, protein-protein interaction (PPI) indicates that HNRNPA1, SERPINE1 are hub proteins. Once their expression alters, it can impose vast changes on other molecular function. Based on this approach, ethanol may target same pathways that are related to cancer onset. In addition, some epidemiologic studies proved that ethanol consumption is related to increment of cancer risk. Therefore, more investigation is required in this regard to elicit the feasible relationship.

## Introduction

Ethanol can contain cytotoxic effect in human body (1, 2). According to studies, many health impairments, chronic diseases, and death around the world is associated with ethanol intake (3, 4). The common kinds are tuberculosis, diabetes mellitus, alcohol use disorders, unipolar depressive disorders, epilepsy, hypertensive heart disease, ischaemic heart disease (IHD), ischaemic and haemorrhagic stroke, conduction disorders and other dysrhythmias, lower respiratory infections (pneumonia), cirrhosis of the liver, preterm birth complications, alcoholic liver ,decrease fertility and fetal alcohol syndrome (1), and medication interactions (5, 6). Furthermore, ethanol consumption has been shown to have a link to cancer onset (7). This is reported in many epidemiological researches (8-10). Mouth, nasopharynx, other pharynx and oropharynx cancer, oesophageal cancer, colon and rectum cancer, liver cancer, and female breast cancer are the some of the known malignancies that are related to polymorphisms in genes implicated in ethanol metabolism. However, the exact underlying molecular mechanism is yet to be recognized (1, 3). Studying molecular layers of biological component introduces us better understanding of how organisms function (11). Proteins as the motor functional parts in human body have a indispensible role (12, 13). Molecular biology indicates that proteins are in the close interaction with other vital molecules including DNA and RNA. These relations mediate metabolic and signaling pathways, cellular processes, and organismal systems. Expression profile of proteins with altered expression can be asses by proteomics (14, 15). This method is a promising application to evaluate proteins in large-scale pattern (16, 17). In fact, significant information related to over-expression or under-expression of designated proteome can be achieved by proteomics (18). In addition, through in-silico experiments, it is possible to decipher molecular basis and relations of different kinds of diseases (19). Examining protein interactions can provide deep insight in biological and biomedical research (1). It is now established that different kinds of complex multi-genetic diseases such as cancer and autoimmune disorders pathogenicity is rooted in complex integration of set of proteins and other molecules (20, 21). Thus, the diseases onset and their progression mechanism can be understood by protein network analysis, in which can provide appropriate diagnostic and therapeutic approaches. Based on recent studies, ethanol showed containing cytotoxic and proteome alteration properties in 270mM dosage (1). Considering this, it is important to examine the proteome alteration concerning candidate proteins in the presence of ethanol. Investigation of protein-protein interaction network and analysis of some proteins involved in ethanol consumption in high dose is the subject of this research. 

## Experimental

*Materials*

The human foreskin fibroblast cells (HFFF2) NCBI Code: C163 was obtained from National Cell Bank of Iran, Pasteur Institute of Iran and used for this study. All the proteomics chemicals and Ready Strip™ IPG strips were provided from GE Health Life Science.

*Experimental procedure*

Treated HFFF2 cells with ethanol and normal HFFF2 cells were prepared as previous study procedure (1). After reaching 80% confluency, they lysed with lysis buffer containing 8 M urea, 4% CHAPS (3-(3-cholamidopropyl) dimethylammonio- 1-propanesulfonate), 40 mM dithiothreitol (DTT), 2% pharmalyte (pH 3–10NL), 1 mM phenylmethylsulfonyl fluoride (PMSF), and 1 mM ethylene diamine tetra-acetic acid (EDTA) for 1h and followed by sonication with a probe sonicator for 5 min to complete the lysis. After that, the samples were centrifuged at 40000 g for 30 min and the supernatants containing proteins were collected for next evaluations. Quantification of proteins was handled by Bradford method, and then the samples were stored at -20^o^C till the next step (2D electrophoresis) (1). Isoelectric focusing (IEF) as the first dimension electrophoresis was carried out with 17 cm (pH 3–10NL) IPG strips. Approximately 1mg protein was loaded in to each gel and triplicate gels for each sample were run to achieve reproducible results. Briefly, the strips were rehydrated in the absence of electric field for 4 h and then with 50 V for 8 h. First dimension electrophoresis was performed by Isoelectric focusing (IEF), which was programmed at a gradient mode. It was first focused for 3 h at the different voltages including 500, 1000 and 8000 V, respectively, then continued at 8000 V and finally increased to 50 KVh. The focused strips were equilibrated in buffer with 6 M urea, 50 mM Tris–HCl, 30% glycerol, 2% SDS and trace bromophenol blue, and were subsequently treated by the reduction of DTT and alkylation of iodoacetamide. The treated strips were transferred onto 12% uniform SDS polyacrylamide gels (second dimension of electrophoresis) running in 2.5 W each gel for 30 min and 15 W each gel until the bromophenol blue dye reached the bottom of the gel. The gels were visualized by Coomassie brilliant blue staining and scanned by BioRad Image Scanner. Finally, protein expression alteration analysis was performed by SameSpots software based on above significant score threshold (Fold>2) (1). All the experiments including cell culture, protein assay, and 2DE were repeated three times and mean and standard deviation were considered. Furthermore, statistical and bioinformatics analysis was handled by R software and Cytoscape program respectively. The distances of expression level difference of designated proteins were calculated as a constructed matrix. Then it was used for clustering analysis using R programming language version (3.0.2) download from (http://www.r-project.org/). The hierarchical cluster is calculated based on distance dissimilarity and the relevant matrix (22). In addition, Box-plot was illustrated by R software. Cytoscape 3.2.1 is an open source tool that presents resourceful environment for data visualization in the form of complex networks available on (www.cytoscape.org) (23). By the use of this source, it is tried to identify hub proteins as they are important in network function. Additionally, network analysis was performed and data were tabulated in the related table. Gene ontology is the reference of controlled vocabulary of the terms of three biological annotation including cell compartment (CC), molecular function (MF), and biological process (BP) (20). Gene ontology annotations were retrieved by application of BioMart  (www.biomart.org), This online tool is designed to provide significantly represented GO terms in a set of genes (24). The significant annotation of terms was carried out based on *P*<0.05 cut off. 

## Results

**Figure 1.**
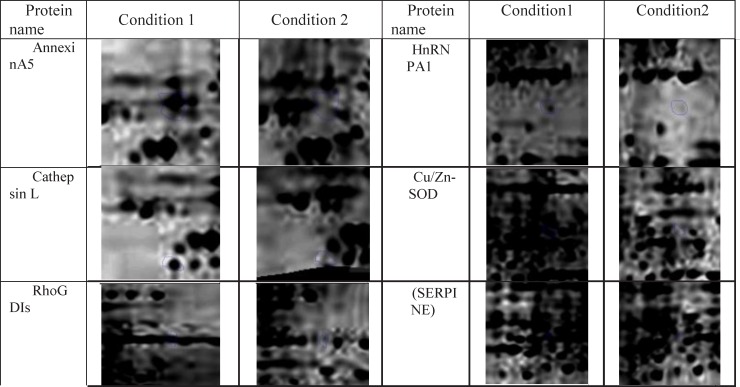
Expression alteration of six indentified proteins in the presence of 270 mM dosage of ethanol is depicted. Conditions 1 and 2 are normal fibroblast gel and ethanol treated gel respectively. These alterations are significant as the Fold>2 and *P*< 0.05

In this research protein expression changes of human fibroblast cells in normal condition and in the presence of ethanol analyzed by 2D gel Electerphoresis and SameSpots software. In Figure 1, six proteins spots with expression alteration are identified. These proteins were recognized via gel comparison from a study conducted by Ge *et al.* (2012) (25). All of these six proteins showed down-regulation after treatment with ethanol. (Figure 1)

For more resolution, a matrix was constructed based on dissimilarity of changes in protein expression among six identified proteins. In a way that, the distance between every pair of proteins was calculated as expression pattern variation and then compared with the rest of proteins (See Table1).

**Table 1:** Constructed distance matrix based on dissimilarity of changes in expression of designated six proteins. Numbers correspond to the proteins (AnnexinA5,1 - Heterogeneous nuclear ribonucleoprotein A1,2 - Cathepsin L,3 - Cu/Zn-SOD ,4 - Rho-GDP dissociation inhibitor ,5 and SERPINE1, 6) The distance between protein 1 and protein 6 is the farthest, whereas the distance between protein 4 and 5 is the closest

**Protein**	**1**	**2**	**3**	**4**	**5**	**6**
1	0.00	0.06	0.10	0.15	0.16	0.20
2	0.06	0.00	0.04	0.09	0.10	0.14
3	0.10	0.04	0.00	0.05	0.06	0.10
4	0.15	0.09	0.05	0.00	0.01	0.05
5	0.06	0.10	0.06	0.01	0.00	0.04
6	0.20	0.14	0.10	0.05	0.04	0.00

**Figure 2:**
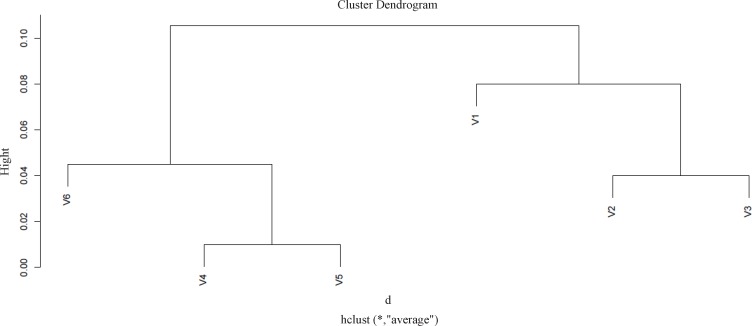
Protein expression profile of six identified proteins based on their similarity to each other by application of R software. Cutting branches off the dendrogram assign clusters. The pairwise including protein V_2_ and protein V_3_ are close in changes in expression and grouped in distinct cluster; protein v4 and protein v5 are the most correlated spots in as grouped in a specific cluster. On the other hand, the v1 and v6 show against changes in expression behavior

While protein clustering is applied for grouping large sets of objects, it can also provide valuable information for small numbers of elements. The applied clustering algorithm is Agglomerative Hierarchical Clustering as it is depicted in Figure 2.

**Figure 3:**
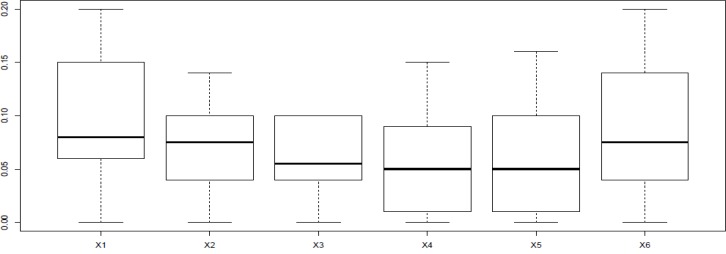
Box-plot obtained from R language software version 3.0.2. The pattern of distribution indicates the changes in expression dissimilarity among six proteins. The box-plots of x_2 _and x_3 _indicate shortest rang comparing to others. Plots, x1 and x6 are the tallest impaling on quite different from other proteins in the terms of expression changes. In addition, changes in expression pattern of x_4 _and x_5_ are more similar

Difference in protein expression profile was plotted by the use of R software (Figure 3)

**Figure 4:**
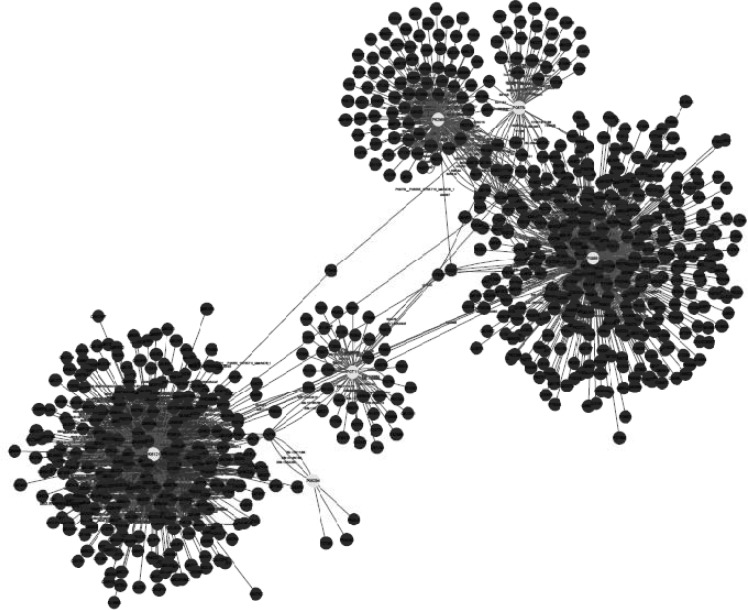
Protein-protein interaction of six proteins involved in ethanol network in which the number of nodes is 796 and edges is 1177. (Designate proteins are shown in highlights). HNRNPA1 with 517 and SERPINE1 with 353 nodes are considered as hub proteins based on degree and betweenness centrality scores

Network visualization of protein-protein interaction was handled by the use of Cytoscape (free open-source software). The integrated network was obtained from Mentha, Reactome-Fls, and STRING Databases by the application of PSICQUIC source, in which nodes of the network represent proteins and their relationship are indicates as edges (Figure 4). The network then analyzed based on degree and betweenness centrality (The data is not shown).

**Figure 5:**
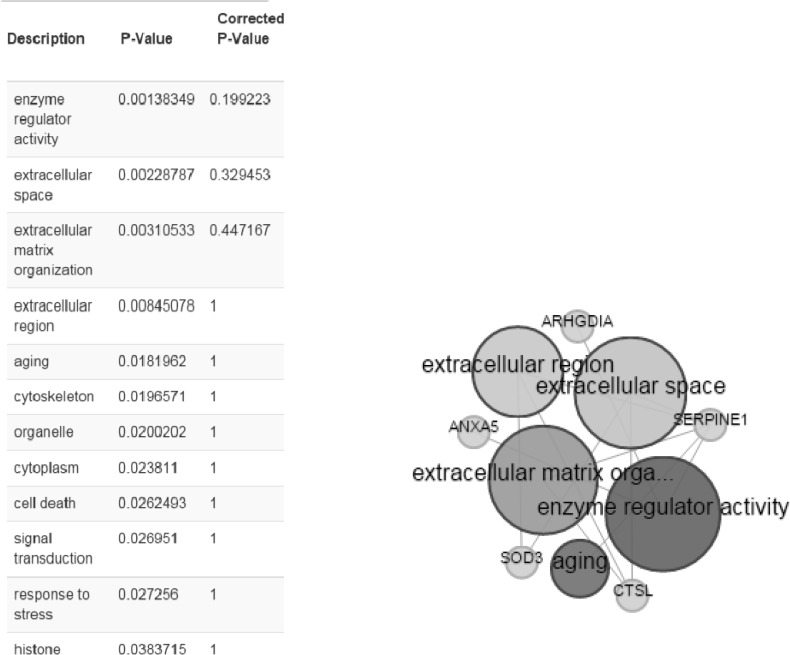
Gene ontology enrichment analysis by the use of BioMart online software. Right, the bigger the circles, the more annotated to the term based on cut off *P*<0.05 and left, statistical properties of annotations

BioMart is applied for gene ontology annotation (www.biomart.org). Here, three different ontology annotation: (MF), (BP), (CC), are enriched for six identified proteins (Figure 5).

## Discussion

As ethanol effects on human fibroblast cells survival and can imposes vast expression alteration on human fibroblast proteome profile (1), it is important to investigate the proteins with significant altered expression and their properties. Based on our previous study, a total number of 65 of proteins belong to fibroblast profile showed significant expression alteration induced by 270 mM dosage of ethanol (1). Among the proteins with significantly expression alteration , six of them were identified by the use of gel comparison from literature (25). They include Annexin A5 (or annexin V), Heterogeneous nuclear ribonucleoprotein A1 (HnRNPA1), Cathepsin L, Rho GDP-dissociation inhibitor (Rho-GDI), Cu,Zn-superoxide dismutase (Cu/Zn-SOD), and Plasminogen activator inhibitor-1 (PAI-1) as it is shown in Figure 1. All of the identified proteins with at least a 2-fold change in their expression are down-regulated. Amazingly, all these six proteins were also evince the same pattern in cancer protein profile changes. It is proved in proteomic analysis of nasopharyngeal carcinoma-associated stromal fibroblasts (25). In addition, previous studies suggest that ethanol consumption can increase the risk of other types of malignancies such as digestive tract cancer, and especially breast cancer (7, 9, 26). Annexin A5 (belongs to annexin family) has a role in cellular signal transduction, inflammation, growth and differentiation. Many diseases are related to the activity of this protein including different types of malignancies, complication of pregnancy, cardiovascular disease, systemic lupus erythematosus, and Ischemia (27-29).  Many kinds of cancers such as pancreatic adenocarcinoma, sarcoma, tumorigenesis and progression of breast cancer and prostate cancer stem cells are linked to Annexin A5 (Anxa5) activity, but its contribution is different in variety of malignancies. In fact, while it has a high expression in cervical cancer, it has a significant low expression in cervical carcinoma cells. It also has a low expression in thyroid cancer. (30).  (HnRNPA1) is a DNA binding protein (31) and its reduction is linked to many kinds of neurodegenerative diseases such as Alzheimer s’ disease (AD), spinal muscular atrophy (SMA), fronto-temporal lobar degeneration (FTLD), amyotrophic lateral sclerosis (ALS), multiple sclerosis (MS), hereditary spastic paraparesis (HSP) and HTLV-I associated myelopathy/tropical spastic paraparesis (HAM/TSP) (32). It is also reported as lung cancer and colon cancer biomarker (33, 34). Cathepsin L involves in progression of the cell cycle regulation via proteolytic processing of the CDP/Cux transcription factor (35). Abnormal expression of this protein is reported for many diseases such as proteinuric kidney disease, atherosclerosis,  Parkinson's disease, and breast cancer (36, 37). Many cellular functions regulate by Rho-GDP dissociation inhibitor (Rho-GDIs). It interacts with Rho family GTPases (38). RhoGDI and GDI/D4 are two mainly known GDIs (39). Cu, Zn-superoxide dismutase (Cu/Zn-SOD) is an antioxidant enzyme in the body. Its copper and zinc ions act as free superoxide radicals destroyer (40, 41). It is related to some diseases such as anxiety, Alzheimer and Parkinson diseases, colon cancer, small cell and non-small cell lung cancer, and gastric cancer (42-46). Serpin peptidase inhibitor clade E (SERPINE1) also known as (PAI-1) as a member of the serpin super-family has a prominent function in regulating the physiological and pathological proteolysis (47). Different diseases are related to dysfunction of PAI-1. One of the main ones is Plasminogen Activator Inhibitor Type 1 deficiency (48). It is known as one of the main regulator of tumor invasion and metastasis regulator, and angiogenesis in Leukemia (49) and other malignancies including cancers of gastric, breast, colon, and ovarian (50-53). As tabulated in table 1, dissimilarity of expression level alteration of proteins, is different from each other. Annexin A5 and SERPINE1 are affected differently as their expression level dissimilarity is higher than the others. On the other hand, Rho-GDP dissociation inhibitor and Cu/Zn-SOD show higher correlation in terms of changes in expression. Hierarchical clustering is a useful method to show the protein spots correlation (22). In addition, box-plot is a standard none-parametric way to depict graphically illustration of sample data variation (54, 55). Here, the visualization and better resolution of expression changes of six proteins based on constructed matrix are shown by dendrogram and boxplot (Figures 2 and 3). However clustering is one of the best methods for analysis of large group proteins and genes but here the significant correlation of two proteins including Cu/Zn-SOD and Rho-GDP dissociation inhibitor can be interpreted by the use of clustering and box-plot methods. Protein-protein interaction is important to analysis and comprehend the relationships between network structure and function (20). It introduces the key hub proteins. Fetching information regarded to interaction profile of evaluated six proteins was from public databases through Cytoscape (Figure 4). Interaction graph indicates that some of the investigated proteins are highly involved in biological associations that are known as hub proteins. The most important ones are HNRNPA1 and SERPINE1. On the other hand, it can also be inferred from protein interaction network that proteins, Cu/Zn-SOD and Rho-GDP dissociation inhibitor and also HnRNPA1 and Cathepsin L with a similar pattern in expression changes are also in close interaction. The first pair is characterized by high degree, but not significant in betweenness centrality. According to BioMart analysis of identified proteins, it is confirmed that extracellular part and enzymatic regulatory activity are the most affected region and biological process respectively in ethanol consumption (Figure 5). SERPINE1 is fundamentally located in extracellular region of cell and plays important roles such as protease binding in which is mainly involved in chronological cell aging and angiogenesis, complement and coagulation cascades and loss of function of SMAD2 and SMAD3 in cancer. Based on annotation, the neighbor proteins of SERPINE1 including TGFB1, TP53, SMAD3, SMAD4 are also involved in cancer (56). It seems that SERPINE1 as a hub protein is involved deeply in cancer so its expression changes may impose a great effect in progress of disease. HNRNPA1 as another hub protein is mainly active in nucleus and acts as nucleotide binding in alternative mRNA splicing process. It is also involved in spliceosome mRNA Splicing. This protein as like SERPINE1 plays a key role in cancer. Therefore, HNRNPA1 and SERPINE1 as hub proteins are involved in cancer and also ethanol consumption; they can be candidate for more investigations. 

## Conclusion

It can be concluded that, there is a similar pattern at least for a part of proteome in cancer and ethanol consumption. This common part includes important proteins that are involved in several key pathways. Since down-regulation of these identified proteins is assigned with cancer and ethanol consumption, they can be consider as biomarker candidate and required to be investigated in more details. 
